# LncRNA *EPR*-induced METTL7A1 modulates target gene translation

**DOI:** 10.1093/nar/gkac544

**Published:** 2022-06-24

**Authors:** Paola Briata, Luca Caputo, Ettore Zapparoli, Elisa Marcaccini, Mario Passalacqua, Lorenzo Brondolo, Domenico Bordo, Annalisa Rossi, Chiara Nicoletti, Gabriele Bucci, Pier Lorenzo Puri, Alberto Inga, Roberto Gherzi

**Affiliations:** Gene Expression Regulation Laboratory, IRCCS Ospedale Policlinico San Martino, 16132 Genova, Italy; Development, Aging and Regeneration Program, Sanford Burnham Prebys Medical Discovery Institute, La Jolla, CA 92037, USA; Center for Omics Sciences, IRCCS Ospedale, San Raffaele, 20132 Milano, Italy; Gene Expression Regulation Laboratory, IRCCS Ospedale Policlinico San Martino, 16132 Genova, Italy; Department of Experimental Medicine (DIMES), University of Genoa, 16132 Genoa, Italy; Inter-University Center for the Promotion of the 3Rs Principles in Teaching & Research (3R Center), 56122 Pisa, Italy; Gene Expression Regulation Laboratory, IRCCS Ospedale Policlinico San Martino, 16132 Genova, Italy; Gene Expression Regulation Laboratory, IRCCS Ospedale Policlinico San Martino, 16132 Genova, Italy; Department of Cellular, Computational and Integrative Biology (CIBIO), University of Trento, Trento, Italy; Development, Aging and Regeneration Program, Sanford Burnham Prebys Medical Discovery Institute, La Jolla, CA 92037, USA; Center for Omics Sciences, IRCCS Ospedale, San Raffaele, 20132 Milano, Italy; Development, Aging and Regeneration Program, Sanford Burnham Prebys Medical Discovery Institute, La Jolla, CA 92037, USA; Department of Cellular, Computational and Integrative Biology (CIBIO), University of Trento, Trento, Italy; Gene Expression Regulation Laboratory, IRCCS Ospedale Policlinico San Martino, 16132 Genova, Italy

## Abstract

*EPR* is a long non-coding RNA (lncRNA) that controls cell proliferation in mammary gland cells by regulating gene transcription. Here, we report on *Mettl7a1* as a direct target of *EPR*. We show that *EPR* induces *Mettl7a1* transcription by rewiring three-dimensional chromatin interactions at the *Mettl7a1* locus. Our data indicate that METTL7A1 contributes to *EPR*-dependent inhibition of TGF-β signaling. METTL7A1 is absent in tumorigenic murine mammary gland cells and its human ortholog (METTL7A) is downregulated in breast cancers. Importantly, re-expression of METTL7A1 in 4T1 tumorigenic cells attenuates their transformation potential, with the putative methyltransferase activity of METTL7A1 being dispensable for its biological functions. We found that METTL7A1 localizes in the cytoplasm whereby it interacts with factors implicated in the early steps of mRNA translation, associates with ribosomes, and affects the levels of target proteins without altering mRNA abundance. Overall, our data indicates that METTL7A1—a transcriptional target of *EPR*—modulates translation of select transcripts.

## INTRODUCTION

We have recently identified *EPR* (acronym for Epithelial Program Regulator, ENSMUST00000098713.5), an intergenic lncRNA highly enriched in epithelial tissues (1). Although *EPR* contains an open reading frame that is translated into a small peptide localized at epithelial cell junctions, we reported that this lncRNA profoundly affects gene expression in mammary gland cells with most changes being independent of the peptide biogenesis (1). We found that *EPR* overexpression prevents TGF-β-induced Epithelial to Mesenchymal Transition (EMT) and inhibits cell proliferation in immortalized mammary gland cells (1). We investigated this last aspect in detail and proved that *EPR* regulates the expression of *Cdkn1a*—the gene encoding the cyclin-dependent kinase inhibitor CDKN1A (p21WAF1/Cip1)—at both transcriptional and post-transcriptional levels (1). Importantly, *EPR* expression in transformed mammary gland cells restrains cell proliferation and migration and it impairs breast tumor formation in an orthotopic transplant model in mice (1). We demonstrated that *EPR* directly binds to chromatin and then—by integrating data derived from ChIRP-Seq (chromatin isolation by RNA purification followed by high-throughput sequencing), ChIP-Seq as well as RNA-Seq in a comprehensive analysis—identified a group of *bona fide* direct transcriptional *EPR* targets (2).

We wanted to investigate the connection between the transcriptional regulation exerted by *EPR* and the phenotypic changes induced by the lncRNA. To this purpose, among genes whose expression is enhanced by *EPR* through direct interaction with promoter/enhancer regions, we focused on *Mettl7a1* (*METTL7A* in human where is also known as AAM-B) which has been proposed to encode a putative *S*-adenosyl methionine (SAM)-dependent methyltransferase (3). Human METTL7A was described as an integral membrane protein anchored into the endoplasmic reticulum (ER) where it recruits cellular proteins for lipid droplet formation (3,4). METTL7A is downregulated in primary thyroid cancers and transformed thyroid cell lines compared to controls (5) and has been proposed to be a tumor suppressor in hepatocellular carcinoma (6). Mellor and colleagues demonstrated that RHOBTB1 depletion causes reduced Golgi integrity through METTL7B —a molecule very similar to METTL7A—and that METTL7A overexpression can compensate for METTL7B activity (7). Further, METTL7A promotes osteogenic differentiation under metabolic stress (8) and odontogenic differentiation (9). Biddy and colleagues reported that murine METTL7A1 is upregulated along with the reprogramming trajectory of mouse stem cells (10). In general, METTL7A is viewed as a factor involved in cell differentiation and tumor suppression even though one report has described METTL7A as an inducer of methotrexate resistance through activation of pro-survival signaling pathways in choriocarcinoma cells (11). Murine *Mettl7a1* has been poorly studied.

Here, we report that *Mettl7a1* is a direct transcriptional target of the lncRNA *EPR* and its induction is mediated by *EPR*-dependent rewiring of chromatin interactions at *Mettl7a1* locus. Importantly, METTL7A/METTL7A1 was found downregulated in human breast cancers and absent in tumorigenic murine mammary gland cells. *Mettl7a1* re-expression in 4T1 cancer cells reduces their transformation potential independently of the putative methyltransferase activity. Mechanistically, METTL7A1 interaction with RNA binding proteins and translation factors leads to selective mRNA translation.

## MATERIALS AND METHODS

### Cell lines, plasmids, siRNAs and transfections.

Murine immortalized NMuMG cells (ATCC, no. CRL-1636) were cultured in DMEM plus 10% FBS and 10 μg/ml bovine insulin (Sigma-Aldrich). NMuMG cells stably over-expressing full-length *EPR* (from nucleotide 1 to 1487 of murine BC030870, NMuMG-*EPR*) as well as cells stably transfected with the empty vector (mock) have been previously described (1) and were maintained in selective medium containing 800 μg/ml G418 (Sigma-Aldrich). For some experiments NMuMG-mock and NMuMG-*EPR* cells were maintained in DMEM supplemented with 2% for 16 h prior to the addition of 5 ng/ml human recombinant TGF-β1 purchased from R&D Systems. 4T1 mouse mammary gland cancer cells (obtained from ATCC, no. CRL-2539) were cultured in DMEM/F12 plus 10% FBS. NMuMG and 4T1 cells were stably transfected with full-length *Mettl7a1* cDNA cloned into pBICEP-CMV-2 vector using GenJet or Lipofectamine 3000 (SignaGen Laboratories and Thermo-Fisher) respectively and were maintained in selective medium containing 800 and 350 μg/ml G418 (SigmaAldrich), respectively. For some experiments, NMuMG cells were transiently transfected with either empty pCMV6-Ac-Myc-His (mock) or with full-length *Mettl7a1* cDNA cloned into the same vector. NMuMG cells were also transiently transfected with either empty pCMV6-Ac-Myc-His (mock) or with full-length murine *Hspa5* cDNA cloned into the same vector. METTL7A1(D98A) mutant was obtained by Site-Directed Mutagenesis of METTL7A1 expression vector using the QuikChange II mutagenesis kit (Agilent Technologies) and the oligonucleotide 5′-TCGGGGAAGCTAACTCTGCTGGAGGTGGGC-3′. Human osteosarcoma cells U2OS cells were obtained from the Biological Resource Center ICLC Cell bank at the IRCCS Ospedale Policlinico San Martino. siRNAs used to knockdown *EPR* were purchased by ThermoFisher (Assay IDs: n516913, n516914, n252093 and n252088) and utilized independently at the concentration of 10 nM each obtaining similar results. siRNA ID. n516913 was routinely used for most experiments. siRNAs used to knockdown *Mettl7a1* were purchased by ThermoFisher (Assay IDs: s206283, s206282 and s88576) and utilized independently at the concentration of 10 nM each obtaining similar results. siRNA ID. s206283 was routinely used for most experiments. Control siRNA was from Santa Cruz Biotechnology (sc-37007).

### Antibodies and cell extracts preparation

The antibodies used in this study are listed in Supplementary Table S1.

Cell extracts were prepared in lysis buffer (50 mM Tris–Cl, pH 8.0, 0.5% Triton X-100, 150 mM NaCl supplemented with complete protease and phosphatase inhibitors (Roche)). When indicated, cell extracts were pre-treated for 30 min at 37°C with RNase A (100 μg/ml).

### qRT-PCR and analysis of nascent transcripts

Total RNA was isolated using the TriPure reagent (Roche) and retro-transcribed (100 ng) using Transcriptor Reverse Transcriptase (Roche) and random hexamers according to manufacturer's instructions. Quantitative PCR was performed using the Luna Universal qPCR master mix (NEB), and the Realplex II Mastercycler (Eppendorf) according to manufacturer's instructions. The sequence-specific primers utilized for PCR reactions are listed in Supplementary Table S2 and have been synthesized by TIB MolBiol (Genova). To analyze gene expression changes among the pool of nascent mRNAs, we adopted the Click-It^®^ Nascent RNA Capture kit (ThermoFisher) and performed experiments according to manufacturer's instructions. Either NMuMG-*EPR* or mock cells were pulsed with 0.5 mM 5-ethynyl Uridine (EU) for 1 hour, clicked, retrotranscribed, and analyzed by qRT-PCR according to Click-iT Nascent RNA Capture kit instructions.

### RNA deep-sequencing (RNA-Seq) and RNA-Seq analysis

High-quality RNA was extracted from either mock or METTL7A1-overexpressing 4T1 cells (biological triplicates for each experimental condition), and a total of six libraries were prepared using standard Illumina Stranded Total RNA Prep with Ribo-Zero Plus protocol and sequenced on Illumina NovaSeq 6000. Image analysis and base calling were performed using the Illumina NovaSeq Control Software. This approach yielded between 30 and 70 million reads that were further processed. Raw reads were trimmed at the ends to remove low-quality calls with BBDuk v38.49 from BBTools suite (BBMap–Bushnell B.–sourceforge. net/projects/bbmap/). Paired-end reads were aligned and mapped to indexed mm10 genome with STAR v2.5.3a. R v3.5.1 and Bioconductor v3.8 were used for secondary analysis. featureCounts function from Rsubread v1.6.4 package was used to assign read counts to the genes of the Ensembl GRCm38.97 gene annotation. Only transcript with at least 1 cpm (counts per million) in at least three samples were considered. We kept mock vs METTL7A1 differentially expressed transcripts when the observed Bayesian statistic was significant (Benjamini and Hochberg corrected *P* value < 0.01; |log FC| > 1.5).

### Ribonucleoprotein complexes immunoprecipitation (RIP) assays

Briefly, total cell lysates were immunoprecipitated with Dynabeads (Thermo Fisher) coated with protein A/protein G and pre-coupled to specific antibodies at 4°C overnight. Pellets were washed three times with a buffer containing 50 mM Tris–HCl (pH 8.0), 150 mM NaCl, 0.5% Triton X-100, 1× Complete (Roche) (1). Total RNA was prepared from immunocomplexes using the QIAzol Lysis Reagent, retrotranscribed, and amplified by qPCR as described above. The primer sequences are listed Supplementary Table S2.

### m6A RNA Quantification

m6A RNA quantification was performed using the colorimetric EpiQuik m6A RNA methylation quantification kit (Epigentek).

### MeRIP analysis

Total RNA was extracted using Trizol reagent (Thermo-Fisher) following the manufacturer's procedure. The total RNA quality and quantity were analysed using the Bioanalyzer 2100 and RNA 6000 Nano LabChip Kit (Agilent). Poly(A)^+^ RNA was purified and fragmented into ∼100-nt-long oligonucleotides using divalent cations under elevated temperature. Then the cleaved RNA fragments were incubated for 2h at 4°C with m6A-specific antibody (No. 202003, Synaptic Systems) in IP buffer (50 mM Tris-HCl, 750 mM NaCl and 0.5% Igepal CA-630) supplemented with BSA (0.5 μg/ml). The mixture was then incubated with protein-A beads and eluted with elution buffer (1 × IP buffer supplemented with 6.7 mM m6A). Eluted RNA was precipitated with 75% ethanol. cDNA libraries were constructed from eluted m6A-containing RNA fragments and from untreated input control fragments. The average insert size for the paired-end libraries was ∼100 ± 50 bp. Library sequencing was performed on an Illumina Novaseq™ 6000 platform at the LC-BIO Bio-tech ltd (Hangzhou, China) following the vendor's recommended protocol.

### m6A-Seq data analysis

First, Cutadapt and perl in house scripts were used to remove the reads that contained adaptor contamination, low quality bases, and undetermined bases. Then sequence quality was verified using FastQC (http://www.bioinformatics.babraham.ac.uk/projects/fastqc/). We used bowtie (http://bowtie-bio.sourceforge.net/index.shtml) to map reads to the genome of *Mus musculus* (Version: v101) with default parameters. Mapped reads of IP and input libraries were analyzed using the R package exomePeak (https://www.bioconductor.org/packages //2.13/bioc/html/exomePeak.html), which identifies m6A peaks in bed or bam format that can be adapted for visualization on the UCSC genome browser or IGV software (http://www.igv.org/). Called peaks were annotated by intersection with gene architecture using ChIPseeker (https://bioconductor.org/packages/devel/bioc/vignettes/ChIPseeker/inst/ doc/ChIPseeker.html). Then StringTie (https://ccb.jhu.edu/software/stringtie/) was used to perform expression level for all mRNAs from input libraries by calculating FPKM (FPKM = [total_exon_fragments/mapped_reads(millions) × exon_length (kB)]). The differentially expressed mRNAs were selected with |log_2_ FC| ≥ 1.5 and *P* value <0.01 by R package edgeR (https://bioconductor.org/packages/release/bioc/html/edgeR.html).

### Immunofluorescence

Mock cells (either NMuMG or 4T1) or their stable transfectants overexpressing FLAG-tagged METTL7A1 were plated on glass chamber-slides (60 000 cells/well). Immunofluorescence was carried out 2 days after plating essentially as reported in (1). Rabbit polyclonal anti-METTL7A antibody (Abcam) and anti-FLAG antibody (F1804, Sigma) were used at a 1:500 dilution. Secondary antibodies, Alexa Fluor 488 anti-rabbit (Thermo Fisher) dilution 1:400, Alexa 488 anti-mouse (ThermoFisher) dilution 1:400, were diluted in IF buffer and incubated for 30 min at 37°C. ER Tracker dye for endoplasmic reticulum labeling was from Molecular Probes (E34251). Nuclei were counterstained with TO-PRO-3 (ThermoFisher). Images were collected using a three-channel TCS SP2 laser-scanning confocal microscope (Leica Microsystems). Images were imported into ImageJ to split and merge channels, cropped and adjusted for resolution and for intensity level range using Photoshop (scale bar = 10 μm).

### Phalloidin staining

4T1 mock and 4T1-METTL7A1 cells were fixed in 4% formaldehyde, stained using Alexa Fluor 568 Phalloidin (ThermoFisher), and imaged with the TCS SP2 laser-scanning confocal microscope (Leica Microsystems).

### Clonogenic assay

The clonogenic (or colony forming) assay was performed essentially as summarized below. Mock cells (either NMuMG or 4T1) or their stable transfectants overexpressing FLAG-tagged METTL7A1 were plated in 6-well multi-wells plates (in sextuplicate). The number of cells plated has been established based on pilot experiments conducted in mock-transfected cells to obtain from 15 to 100 colonies per well after at least six replication times. Colonies were stained with 2 ml 0.01% (w/v) crystal violet in H_2_O for 30 min and counted using the imaging analysis software package ImageJ 1.53a (http://imagej.nih.gov/ij, NIH).

### Cell migration assay

Mock cells (either NMuMG or 4T1) or their stable transfectants overexpressing FLAG-tagged METTL7A1 were cultured in six-wells plates up to confluence (in sextuplicate) and pretreated for 2 h with 5 μg/ml Mitomycin C (Sigma-Aldrich). A wound was scratched into monolayers and cells were cultured for up to 48 h in the presence of 5 μg/ml Mitomycin C. Images were taken using an Olympus CKX41 microscope and analyzed using the ImageJ 1.53a package. Average distance of wound obtained from six microscopic fields was used for the calculation of percent wound healed. Experiments were performed three times in triplicate.

### Extracellular matrix invasion assay

The assay was conducted using the Cell Invasion Assay kit (ECM550 from EMD Millipore) according to manufacturer's instructions. After staining, images were taken using an Olympus CKX41 microscope and migrated cells were counted using the imaging analysis software package ImageJ 1.53a.

### Anchorage-independent cell growth

Anchorage-independent cell growth assays were assessed according to the protocol described in (1). Briefly, 2500 cells were seeded in 0.3% top agar in complete medium and placed on a layer of 0.5% of bottom agar in 12-well plates. Each cell line was seeded in sextuplicate and fed every 3 days. After 21 days, cells were colored with crystal violet and photographs were taken.

### Proteasome activity assay

The proteasome activity assay was carried out using the 20S Proteasome Assay Kit (Cayman Chemical). 4T1-mock and 4T1-METTL7A1 cells were cultured in 96-well plates and tested when confluency was near 90%, usually 24 h after seeding. Cells were washed twice with 20S proteasome assay buffer and lysed with 100 μl of the provided lysis buffer using an orbital shaker at room temperature for 30 min. 90 μl of the lysate was then transferred to a black flat-bottom 96-well plate and 10 μl of assay buffer or 10 μM Ixazomib (MNL2238), an inhibitor of chemotrypsin-like activity of the β5 subunit of the 20S proteasome, were added to respective wells to measure activity or test assay specificity. Enzymatic activity was quantified using a plate reader by measuring fluorescence units emitted at 480 nm (excitation at 360 nm) resulting from cleavage of the specific substrate SUC-LLVY-AMC provided.

### Click-iT^®^ metabolic labeling of proteins

4T1-mock and 4T1-METTL7A1 cells were labelled for 210 min with l-azidohomoalanine (AHA, Invitrogen). Extracts were prepared and Biotin Click-iT^®^ reactions were performed exactly as described in Manufacturer's instructions. Then ‘Clicked’ reactions were diluted and precipitated (overnight at 4°C rotating) with Streptavidin beads (MyONE-Streptavidin C1, Invitrogen). After extensive washes, proteins were eluted from beads with 1× SDS-Sample Buffer and analyzed by SDS-PAGE and Immunoblotting.

### CRISPR-Cas9 experiments

Two Guide RNA (gRNA) targeting the removal of the whole exon 1 (993 bp) of *Mettl7a1*-201 and 204 isoforms [ENSMUST00000067752.5 and ENSMUST00000229588.2, respectively] were designed, generated, and evaluated for their absence of off-target activity and ability to mediate cleavage and indel formation (Metttl7a1-g.: 5’-GCCGGGTAGGGCTCCGATTG-3’; Metttl7a1-g2: 5’-AAAACCTTCCAAGTCCCGGC-3’). NMuMG cells were transfected using Neon transfection system (Thermo Fisher). Genomic DNA was extracted from pooled cells and PCR tests were performed to evaluate the presence of the 5’ arm and the 3’ arm before and after editing as well as the presence of exon 1. Pools were subjected to single cell cloning via manual seeding (limiting dilution) into 96-well plates. Single cell clones were grown for about 20 days, DNA was extracted, and PCR was used to identify potential knock-out (KO) clones. PCR products from the heterozygous clones were purified and sequenced using next-generation sequencing to confirm the results.

### Analysis of METTL7A expression in human samples

To investigate the expression of METTL7A in cancer samples, we interrogated publicly available proteomic datasets from the Clinical Proteomic Tumor Analysis Consortium (CPTAC, NIH) using the interactive web resource UALCAN (12).

### *In situ* 3C analysis (a.k.a. 4C)

3C-Seq analysis was performed as previously described (13) with some modifications. Trypsinized cells were cross-linked in suspension with 2% formaldehyde for 10 min at room temperature in culture medium. Formaldehyde was then quenched with 200 mM of glycine for 5 min at 4°C. Cells were then washed in ice-cold PBS, pelleted, and lysed with lysis buffer (10 mM Tris–HCl pH 8.0, 10 mM NaCl, 0.2% NP40, 1× cOmplete [Roche]) for 10 min at 4°C. The nuclear pellets were washed once with PBS, snap-frozen, and stored at –80°C. Nuclei were incubated with 0.5% SDS in ultrapure H_2_O at 62°C for 10 min in a Thermomixer (600 rpm, Eppendorf) then SDS was quenched with Triton X-100 for 15 min at 37°C with shaking (600 rpm). Restriction Enzyme buffer (NEBuffer, New England Biolabs) was added to reach 1× final concentration. DNA was then digested with 600U *DpnII* restriction endonuclease (New England Biolabs) overnight at 37°C at 900 rpm. Inactivation of *DpnII* was performed by incubating the samples at 62°C for 20 min at 900 rpm. Digestion was checked by agarose gel electrophoresis and ligation with T4 DNA Ligase (New England Biolabs) was carried out for 4 h at 20°C at 900 rpm. DNA was then extracted with Phenol–chloroform–isoamyl alcohol mixture (Sigma Aldrich) and Ethanol precipitated. Ligation efficiency was checked by agarose gel electrophoresis. DNA (80 μg) was then digested with 80U *BfaI* restriction endonuclease (New England Biolabs) overnight at 37°C, extracted with Phenol–chloroform–isoamyl alcohol, Ethanol precipitated, and re-ligated with T4 DNA ligase (overnight at 16°C). After further phenol–chloroform–isoamyl alcohol extraction and Ethanol precipitation, DNA was resuspended in 10 mM Tris–HCl pH 8.0 and purified using the High Pure PCR purification kit (Roche). 3C-Seq libraries construction with Illumina primers was performed as described in Stadhouders *et al.* (13).

### 3C-Seq data analysis

3C-Seq libraries were sequenced on Illumina NextSeq 500 in single-end and 75 cycle mode. Reads were processed with pipe4C v1.0 pipeline (14) using UCSC mm10 reference genome, chr15:100,302,780 as viewpoint, and CAGCAACCACATGGTGGCTCA sequence as primer. Peaks were then called using PeakC v0.2 R package (15) with a window size of 10 (wSize = 10), i.e. the number of fragments over which the averaging is performed. ‘Cis’ peaks were selected within 200kb from the viewpoint.

### 3C-PCR

3C-PCR experiments were conducted as described in Hagege *et al.* (16). The sequence of primers used is presented in Supplementary Table S2.

### Sucrose-gradient fractionation and polysome profiling

Experiments were performed as described (1). Either 4T1 mock or 4T1-METTL7A1 cells (∼70% confluence) were treated with cycloheximide (0.1 mg/ml) for 5 min at 37°C, washed twice with PBS supplemented by 0.01 mg/ml cycloheximide, scraped in PBS 1× with 0.01 mg/ml cycloheximide, pelleted by centrifugation, lysed in 500 μl of ice-cold Lysis Buffer (salt solution 1×, 1% Triton-X100, 1% NaDeoxycholate, 0.2 U/μl RNase inhibitor, 1 mM DTT, 0.01 mg ml − 1 cycloheximide), centrifuged for 5 min at 16 000 × g at 4°C, and supernatants were loaded onto sucrose gradients. One ml fractions were collected monitoring the absorbance at 260 nm using a Density Gradient Fractionation System by Teledyne ISCO with sensitivity set to 0.2. Using the profile of the 260 nm absorbance, fractions corresponding to free ribosomal subunits (40S and 60S) and monosomes (80S, considered as not translating), separately from fractions corresponding to light polysomes (2–5 ribosomes) and heavy polysomes (>6 ribosomes) were pooled together for RNA extraction (see below). Proteins were isolated from each fraction through TCA/acetone precipitation. The same volume of 100% ice-cold acetone and 1/10th of TCA was added to each fraction. The samples were placed at –80°C overnight and then centrifuged at 16 000 × g for 10 min at 4°C. Pellets were washed three times with 1 ml of 100% ice-cold acetone, dried at RT for 5 min and resuspended in SDS-PAGE loading buffer.

### Analysis of mRNA distribution profiles in polysome fractions

RNA was extracted from either separate polysome fractions or from pooled fractions (see above) prepared from either 4T1 mock or 4T1-METTL7A1 cells as described in Panda *et al.* (17), retrotranscribed and subjected to qPCR analysis as described above using the transcript-specific primers listed in Supplementary Table S2.

### *In silico* modeling

The model of the METTL7A1 methyltransferase domain was obtained through the web tool Modbase (https://salilab.org/modbase), based on the crystal structure of the SAM dependent methyltransferase from *Pyrococcus horikoshii* (PDB: 1ZWN, 20% amino acid identity with METTL7A1).

### nLC–ESI–MS/MS analysis and Identification of METTL7A1 molecular partners

To identify molecular partners of METTL7A1, 2 mg of total extracts from either mock or NMuMG-Mettl7a1 were immunoprecipitated with anti-FLAG antibody and the immunocomplexes separated by SDS-PAGE electrophoresis and silver stained. Every entire lane was cut in three slices and submitted to a standard in-gel protocol digestion. Reduction with 10-mM DTT, alkylation with 55-mM iodoacetamide, and trypsin digestion were carried out as previously reported (18). After acidification with 0.1% formic acid, peptide mixtures were concentrated and desalted on homemade StageTips C18 (19). Peptides were injected on an UPLC EASY-nLC 1000 (Thermo Scientific) and separated on a 25 cm fused-silica emitter of 75 μm inner diameter (New Objective, Inc. Woburn, MA, USA), packed in-house with ReproSil-Pur C18-AQ 1.9 μm beads (Dr Maisch Gmbh, Ammerbuch, Germany) using a high-pressure bomb loader (Proxeon, Odense, Denmark) (18).

A gradient of eluents A (2% acetonitrile, 0.1% formic acid) and B (80% acetonitrile with 0.1% formic acid) was used to achieve separation, from 5 to 100% B (in 30 min, 250 nl/min flow rate). The nLC system was connected to a quadrupole Orbitrap QExactive-HF mass spectrometer (ThermoFisher) equipped with a nanoelectrospray ion source (Proxeon Biosystems). MS data were acquired using a data-dependent acquisition (DDA) top 15 method with HCD fragmentation. Survey full scan MS spectra (300–1750 Th) were acquired in the Orbitrap with 60 000 resolution, AGC target 1e6, and IT 120 ms. For HCD spectra, resolution was set to 15 000 at *m*/*z* 200, AGC target 1e5, and IT 120 ms: NCE 28% and isolation width 3.0 *m*/*z*. Injection volume was set at 4 μl. Raw data were processed with Proteome Discoverer (version 1.4.1.14, Thermo Scientific) and Mascot (version 2.6.0, Matrix Science) searching against the UniProtKB Mouse complete proteome database (80894 entries), assuming a fragment ion mass tolerance of 0.020 Da and a parent ion tolerance of 10 ppm; the specified enzyme was trypsin with up to 2 missed cleavage allowed; carbamidomethylation of cysteine was set as fixed modifications; oxidation of methionine and N-terminal acetylation were set as variable modifications. Scaffold (version 4.4.3, Proteome Software Inc.) was used to validate MS/MS-based peptide and protein identifications. Peptide identifications were accepted when probability, calculated using Scaffold Local FDR algorithm, was greater than 95%. Protein identifications were accepted when probability was >99% and proteins contained at least four identified peptides. We arbitrarily considered only peptides that were either absent in the immunocomplexes from mock cells or, when present, at least four times more represented in NMuMG-METTL7A1 than in mock cells. Proteins that share similar peptides and could not be differentiated based on MS/MS analysis alone were grouped to satisfy the principles of parsimony.

### nLC–ESI–MS/MS analysis and protein quantification

Proteomics analyses were performed on 4T1 cells either mock or overexpressing *Mettl7a1* (in biological triplicates). 15 × 10^6^ cells were lysed in 8 M urea, 100 mM Tris–HCl pH 8 (Urea buffer) and sonicated with BIORUPTOR Pico (Diagenode) (3 cycles: 30 s on/30 s off).

The extracted proteins were quantified by BCA kit (ThermoFisher) and 50 μg of proteins were reduced and alkylated by adding 10 mM TCEP (Thermo Scientific) and 40 mM 2-chloroacetamide (Sigma-Aldrich) in 8 M urea 100 mM Tris pH 8 for 30 min at room temperature. Urea buffer was diluted to 2 M with 100 mM Tris–HCl pH 8 and proteins were digested with trypsin (Trypsin, Sequencing Grade, modified from Roche) (1:50 = enzyme: proteins) overnight at RT. Peptides were acidified to 5% formic Acid (Sigma-Aldrich) and purified on a C18 StageTip. 2 μl of digested sample were injected onto a quadrupole Orbitrap Q-exactive HF mass spectrometer (Thermo Scientific). Peptide separation was achieved on a gradient of eluents A (2% acetonitrile, 0.1% formic acid) and B (80% acetonitrile with 0.1% formic acid) from 0 to 100% B (in 75 min, 250 nl/min flow rate) on UHPLC Easy-nLC 1000 (Thermo Scientific). The LC system was connected to a 25-cm fused-silica emitter of 75 μm inner diameter (New Objective), packed in-house with ReproSil-Pur C18-AQ 1.9 μm beads (Dr Maisch Gmbh) using a high-pressure bomb loader (Proxeon). The mass spectrometer was operated in DDA mode with dynamic exclusion enabled 15 s, MS1 resolution 60 000, MS1 AGC target 3e6, MS1 IT 20 ms, MS2 resolution 15 000, MS2 AGC target 1e5, MS2 IT 80 ms, and MS2 NCE 28%. For each cycle, one full MS1 scan range 300–1650 *m*/*z* was followed by 15 MS2 scans using an isolation window of 2.0 *m*/*z*. Raw MS files were processed with MaxQuant software (1.5.2.8), making use of the Andromeda search engine (20). MS/MS peak lists were searched against the UniProtKB Mouse complete proteome database (80 894 entries) in which trypsin specificity was used with up to two missed cleavages allowed. Searches were performed selecting alkylation of cysteine by carbamidomethylation as fixed modification, and oxidation of methionine, N-terminal acetylation as variable modifications. Mass tolerance was set to 5 and 10 ppm for parent and fragment ions, respectively. A reverse decoy database was generated within Andromeda, and the false discovery rate (FDR) was set to <0.01 for peptide spectrum matches (PSMs). For identification, at least two peptide identifications per protein were required, of which at least one peptide had to be unique to the protein group.

### Quantitative and statistical analysis

Statistical analysis was performed via Perseus platform (version 1.5.1.6) included in the MaxQuant package. LFQ Intensity were *z*-score normalized and missing values were replaced by random numbers drawn from a normal distribution by the function ‘imputation’. *T*-test, Permutation test analysis were performed applying FDR <0.05 or *P* value <0.05 as reported.

All the graphs, calculations, and statistical analyses were performed using GraphPad Prism software version 9.0 for MacOS (GraphPad Software).

## RESULTS

### Transcriptional regulation of *Mettl7a1* gene by *EPR*

Our previous data indicate that *EPR* limits cell proliferation and migration in mammary gland models (1). We chose to focus our studies on *Mettl7a1*, one of the candidate direct transcriptional targets of *EPR* (2), since our meta-analysis of publicly available proteomic datasets showed that human METTL7A is downregulated in breast cancer as well as in several other cancers (Supplementary Figure S1A).

**Figure 1.**
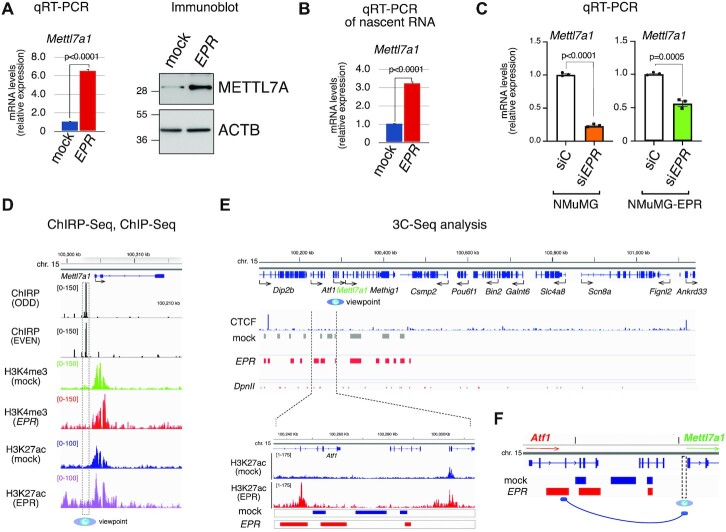
Transcriptional regulation of *Mettl7a1* by *EPR* in NMuMG cells. (**A**) (left) Total RNA was extracted from mock and NMuMG-*EPR* cells and analyzed by qRT-PCR as indicated; (right) total extracts from mock and NMuMG-*EPR* cells were analyzed by SDS-PAGE followed by Immunoblot as indicated. The immunoblots displayed are representative of three experiments that yielded similar results (**B**) qRT-PCR analysis of nascent mRNAs in mock and NMuMG-*EPR* cells. (**C**) qRT-PCR analysis of *Mettl7a1* in either NMuMG or NMuMG-*EPR* transiently transfected with either control siRNA (siC) or siRNA designed to silence *EPR* expression (si*EPR*). The values of qRT-PCR experiments shown are averages (±SEM) of three independent experiments performed in triplicate. Statistical significance (Student's t test) has been calculated using GraphPad Prism 9 for macOS software. (**D**) From top to bottom: genomic coordinates, RefSeq gene from Integrative Genome Viewer (Broad Institute), snapshots of ChIRP-Seq experiments, snapshots of H3K4me3 and H3K27ac ChIP-Seq (2) analyses performed in NMuMG-mock (mock) and NMuMG-*EPR* (*EPR*) centered on *Mettl7a1* gene. The region used as viewpoint for 3C-Seq analysis is surrounded by a vertical dashed box and indicated at the bottom by a blue eye. (**E**) (upper panel), from top to bottom: genomic coordinates, RefSeq genes from Integrative Genome Viewer (Broad Institute), snapshot of CTCF ChIP-Seq (derived from GSM2425116), 3C-Seq bins in NMuMG-mock (mock) and NMuMG-*EPR* (*EPR*) cells, *DpnII* restriction sites. (Lower panel), close-up representation of the region located within the dashed lines. From top to bottom: genomic coordinates centered on the *Atf1* locus, snapshot of H3K27ac ChIP-seq (2) in NMuMG-mock (mock) and NMuMG-*EPR* (*EPR*) cells, 3C-Seq bins in NMuMG-mock (mock) and NMuMG-*EPR* (*EPR*) cells. 3C-Seq analysis was performed using biological duplicates. (**F**) Schematic representation of the proposed *EPR*-mediated recruitment of a putative distal enhancer to the *Mettl7a1* promoter region. 3C-Seq analyses were conducted on biological duplicates.

Both stable and transient full-length *EPR* overexpression in murine immortalized mammary gland NMuMG cells enhances *Mettl7a1* mRNA and protein levels (Figure 1A and Supplementary Figure S1B). Analysis of nascent transcripts revealed that the induction is largely transcriptional (Figure 1B). Importantly, *EPR* silencing in either NMuMG or in NMuMG cells stably overexpressing the lncRNA (NMuMG-*EPR* cells) resulted in a significant reduction of *Mettl7a1* mRNA levels (Figure 1C and Supplementary Figure S1C).

**Figure 2.**
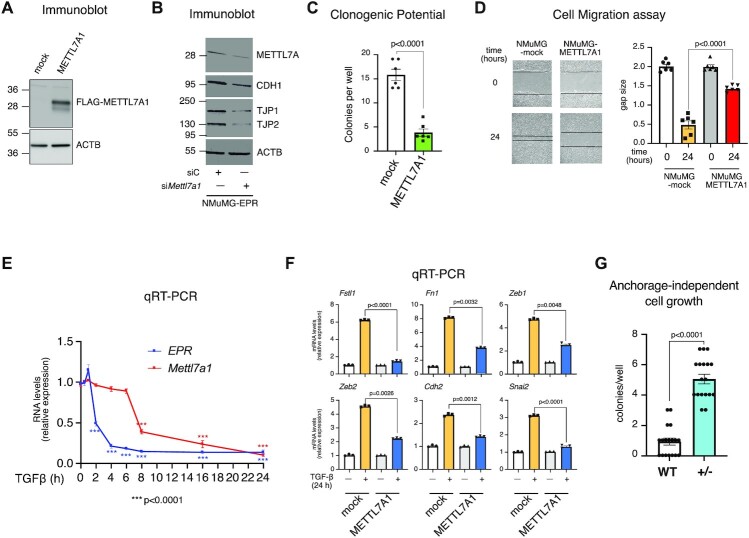
METTL7A1 is a component of an *EPR*-dependent pathway in NMuMG cells. (**A**) Total extracts were prepared from NMuMG cells stably transfected with either the empty vector (mock) or FLAG-tagged METTL7A1 expressing vector (METTL7A1) and analyzed by SDS-PAGE followed by Immunoblot as indicated. (**B**) NMuMG-*EPR* were transiently transfected with either control siRNA (siC) or siRNA designed to silence *Mettl7a1* expression (si*Mettl7a1*), total extracts were prepared and analyzed by SDS-PAGE followed by Immunoblot as indicated. The immunoblots displayed in panels A and B are representative of three experiments that yielded similar results. (**C**) Either mock or NMuMG-METTL7A1 cells were plated at low density in sextuplicate. After 6–10 days colonies were stained and counted using the imaging analysis software package ImageJ 1.53a (http://imagej.nih.gov/ij, NIH). The values are averages (±SEM) of three independent experiments performed in sextuplicate. (**D**) Scratch wound healing assays. (right) Scratch wounds were introduced into confluent monolayers of either mock or NMuMG-METTL7A1 cells. Cultures were photographed and the width of the wound was measured at 0 hr and 24 hr after the scratch was made. (left) The gap area (arbitrary units) for each cell type at each time point was plotted. The values are averages (±SEM) of three independent experiments performed in sextuplicate. (**E**) qRT-PCR analysis of either *EPR* or *Mettl7a1*in NMuMG cells serum-starved (2% FBS, 16 h) and either treated with TGF-β (5 ng/ml) for the indicated times or left untreated (time 0). (**F**) qRT-PCR analysis of the indicated transcripts in either mock or NMuMG-METTL7A1 cells serum-starved and either treated with TGF-β (5 ng/ml) (+) for 24 h or left untreated (−). The values of qRT-PCR experiments shown are averages (±SEM) of three independent experiments performed in triplicate. Statistical significance (Student's *t* test) has been calculated using GraphPad Prism. (**G**). Wild-type NMuMG (WT) and NMuMG heterozygous for *Mettl7a1* CRISPR-Cas9 mediated deletion (+/−) were cultured in soft agar for 21 days and phase contrast micrographs were taken at 10X magnification. The number of colonies was counted and plotted. The values are averages (±SEM) of three independent experiments performed in sextuplicate. Statistical significance (Student's t test) has been calculated using GraphPad Prism 9 for macOS software.

We have demonstrated by ChIRP-Seq analysis that *EPR* binds to the promoter/enhancer region of *Mettl7a1* gene but, intriguingly, this interaction is not accompanied by local enrichment of the histone activation marks H3K4me3 and H3K27ac, differently from most other *EPR* direct targets that we have identified (Figure 1D, (2)). Thus, we hypothesized that *EPR* binding mediates long-range tridimensional arrangements of chromatin that bring some distal enhancer regions in proximity to the *Mettl7a1* promoter. To verify this hypothesis, we performed Chromosome Conformation Capture (3C) analysis coupled to high throughput sequencing (3C-Seq) to characterize the spatial interaction within *Mettl7a1* locus using the *EPR*-bound *Mettl7a1* distal promoter region as viewpoint in mock and NMuMG-*EPR* cells (Figure 1D, (2)). We restricted our analysis to regions contained within two CTCF sites that define the boundaries of Insulated Neighborhoods which typically constrain promoter-enhancer functional interactions (21). Multiple promoter-interacting elements were detected by 3C-Seq in this area, and some showed specific peaks in NMuMG-*EPR* cells (Figure 1E, top panel). Among these, we focused on an intronic region of the *Atf1* gene located approximately 58 kb at the 5’ of *Mettl7a1* transcription start site (magnified in Figure 1E, bottom panel) that displays a strong H3K27ac ChIP-Seq signal in NMuMG-*EPR* cells (Figure 1E, bottom). We confirmed the interaction of *Mettl7a1* promoter with this putative intronic enhancer by both PCR and qPCR (Supplementary Figure S1D). Importantly, *EPR* silencing in NMuMG-*EPR* cells caused a strong reduction of this interaction as revealed by PCR and qPCR analyses (Supplementary Figure S1E).

Altogether our data indicate that *EPR* activates *Mettl7a1* gene transcription by favoring the interactions between *EPR*-bound promoter region and a distal enhancer element (schematic in Figure 1F).

### METTL7A1 is a component of an EPR-dependent pathway in NMuMG cells.

We wanted to investigate the contribution of METTL7A1 to the effects that EPR exerts in NMuMG cells (1). Indeed, overexpression of a FLAG-tagged METTL7A1 in NMuMG cells (NMuMG-METTL7A1 cells, Figure 2A) regulates the mRNA levels of factors involved in cell adhesion and intercellular junctions in a way similar to *EPR* overexpression (Supplementary Figures S2A and B). Accordingly, *Mettl7a1* silencing in NMuMG-*EPR* cells reduces the expression of factors that are upregulated upon *EPR* overexpression (Figure 2B, Supplementary Figure 2C (1)). Further, Figure 2C and D show that NMuMG-METTL7A1 cells display impaired clonogenic potential and migration when compared to mock cells. We also show that, like *EPR*, *Mettl7a1* mRNA levels are rapidly reduced in NMuMG cells upon treatment with TGF-β with *EPR* down-regulation preceding *Mettl7a1* down-regulation by about 4 h (Figure 2E, (1)). In Rossi *et al.* (1), we reported that *EPR* overexpression prevents TGF-β-dependent modulation of factors involved in EMT. Here, we show that forced expression of METTL7A1 in NMuMG cells impairs the TGF-β-induced expression of a group of genes implicated in EMT (Figure 2F, (1)).

We utilized CRISPR-Cas9 technology to knock-out *Mettl7a1* gene in NMuMG cells. Despite repeated attempts with different gRNAs, we could not obtain homozygous knock-out cells, and this suggests that *Mettl7a1* might be an essential gene for NMuMG cells viability. Nevertheless, the phenotype analysis of a representative heterozygous clone revealed that ablation of one allele of *Mettl7a1* results in a significant increase of anchorage-independent growth of NMuMG cells that, being not transformed, do not proliferate in semi-solid media under standard conditions (Figure 2G (22)).

In conclusion, our data suggest that METTL7A1 mediates some of the functions exerted by *EPR* in immortalized mammary gland cells.

### METTL7A1 is a cytoplasmic protein in NMuMG cells and its expression is severely reduced in transformed cell lines

We analyzed cellular localization of METTL7A1 and found that it is located in the cytoplasm of NMuMG cells (Supplementary Figure S3A, B). It has been previously reported that human METTL7A localizes in lipid droplets (3). However, not all cell types possess lipid droplets as shown in NMuMG cells where an antibody directed to Perilipin 2 (anti-PLIN2)—able to coat intracellular lipid storage droplets—did not show any specific staining, contrary to what was observed in U2OS cells (positive control) (Supplementary Figure S3C).

**Figure 3.**
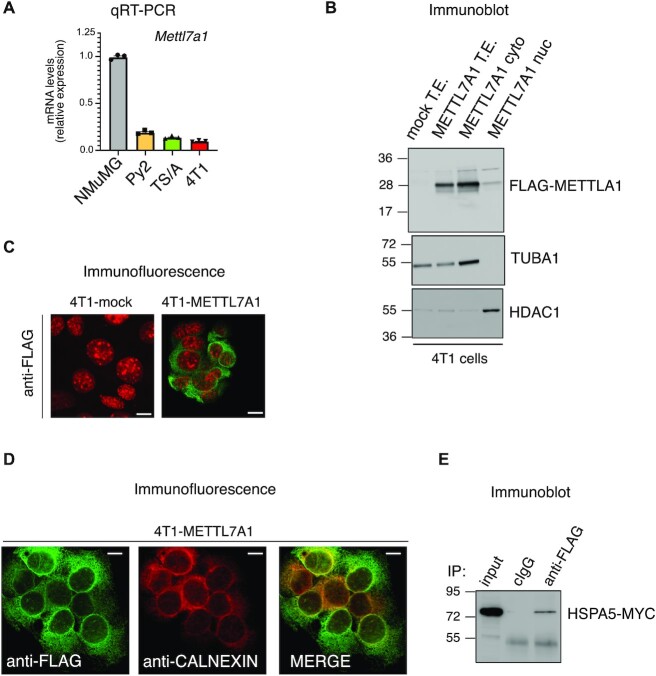
METTL7A1 expression is severely reduced in transformed cell lines. (**A**) The expression of *Mettl7a1* was quantified by qRT-PCR analysis in the indicated cell types. The values of qRT-PCR are averages (±SEM) of three independent experiments performed in triplicate. (**B**) Total, cytoplasmic, and nuclear extracts were prepared from either mock or 4T1-METTL7A1 cells were analyzed by SDS-PAGE followed by Immunoblot using the indicated antibodies. (**C**) Immunofluorescence analysis of either 4T1-mock or 4T1-METTL7A1 cells grown on chamber slides and stained with anti-FLAG antibody. Scale bars represent 50 μm. (**D**) Immunofluorescence analysis of 4T1-METTL7A1 cells grown on chamber slides and stained as indicated. Scale bars represent 50 μm. Immunofluorescence analyses presented in panels (C) and (D) are representative of at least five fields evaluated in two biological replicates. (**E**) Coimmunoprecipitation of FLAG-tagged METTL7A1 expressed in 4T1-METTL7A1 cells transiently co-transfected with MYC-tagged HSPA5. The immunoblots displayed in panels B and E are representative of three experiments that yielded similar results.

According to the evidence that METTL7A is downregulated in human breast cancers (Supplementary Figure S1A), we found that *Mettl7a1* mRNA levels are also strongly reduced in some murine transformed mammary gland cell lines when compared to immortalized NMuMG cells (Figure 3A). We expressed FLAG-tagged METTL7A1 in triple-negative mesenchymal-like mammary gland cancer cells 4T1—in which the protein is virtually absent—(4T1-METTL7A1 cells) and we first observed that also the transfected FLAG-METTL7A1 almost completely localized in the cytoplasm (Figures 3B, C). Further, colocalization with two markers of the ER—calnexin and ER-tracker—revealed that METTL7A1 associated, at least in part, with the ER as confirmed by METTL7A1 co-immunoprecipitation with the ER-resident protein HSPA5 (a.k.a. BiP) (Figure 3D, E, Supplementary Figure S3D).

Altogether our data indicate that both endogenous METTL7A1 and FLAG-tagged transfected METTL7A1 are cytoplasmic proteins associated, in part, with the ER. Importantly, METTL7A1 is not expressed in transformed mammary gland cells.

### METTL7A1 expression in transformed cells impairs their tumorigenic potential.

**Figure 4.**
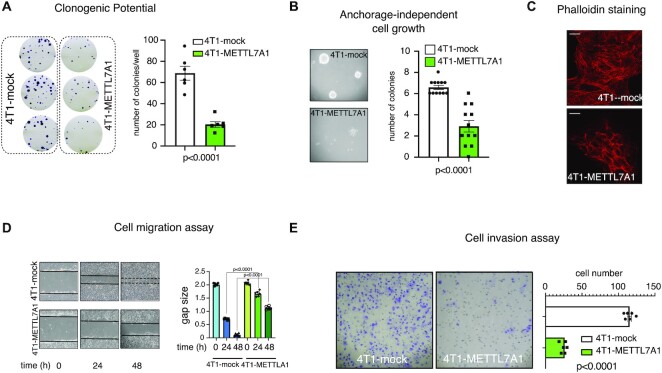
METTL7A1 expression in 4T1 transformed cells impairs their proliferation, migration, and invasion. (**A**) Either mock or 4T1-METTL7A1 cells were plated at low density in sextuplicate. After 6–10 days, colonies were stained and counted using the imaging analysis software package ImageJ 1.53a (http://imagej.nih.gov/ij, NIH). On the left a representative image of stained colonies. The values of three independent experiments performed in sextuplicate (±SEM) are plotted on the right. (**B**) Either 4T1-mock or 4T1-METTL7A1 were cultured in soft agar for 21 days and phase contrast micrographs were taken at 10X magnification. The number of colonies was counted and plotted. A representative image of colonies is shown on the left while the values in the bar graph (on the right) are averages (±SEM) of three independent experiments performed in sextuplicate. (**C**) Either 4T1-mock or 4T1-METTL7A1 were cultured on chamber slides and stained using Alexa Fluor 568 Phalloidin to visualize the actin cytoskeleton. Representative images of at least five fields of each biological duplicate are shown. Scale bars represent 50 μm. (**D**) Scratch wound healing assays. Scratch wounds were introduced into confluent monolayers of either 4T1-mock or 4T1-METTL7A1 cells. Cultures were photographed and the width of the wound was measured at 0, 24 and 48 h after the scratch was made. The gap area (arbitrary units) for each cell type at each time point was plotted. The values are averages (±SEM) of three independent experiments performed in sextuplicate. (**E**) Extracellular matrix invasion assay. 500 000 cells (either 4T1-mock or 4T1-METTL7A1) were seeded in inserts (8 μm pore size polycarbonate membrane coated with extracellular matrix) containing serum-free medium. Inserts were placed in 24-well plates filled with 20% FCS-containing medium. After 48 h, upper cells were removed and migrated cells were stained with Crystal violet and micro-photograms were taken (representative image on the left). Migrated cells were counted and plotted (right). The values are averages (±SEM) of three independent experiments performed in triplicate. Statistical significance (Student's *t* test) has been calculated using GraphPad Prism 9 for macOS software.

We hypothesized that *Mettl7a1* expression in 4T1 cells affects their behavior. We analyzed the phenotype of 4T1-METTL7A1 compared with mock cells and, first, observed that they display a significantly reduced clonogenic potential and an impaired growth ability in semisolid media (Figure 4A, B). Further, Phalloidin staining of mock- and METTL7A1-transfected 4T1 cells revealed different patterns. While mock cells displayed F-actin thick parallel bundles within cells, 4T1-METTL7A1 cells exhibited a pattern of F-actin predominantly organized in cortical bundles, reminiscent of untransformed epithelial cells (Figure 4C). This redistribution could result in altered migration properties and, indeed, our data indicate that METTL7A1 expression significantly reduces the migration potential of 4T1 cells (Figure 4D). Finally, while 4T1 mock cells possess a strong ability of invading extra-cellular matrix, the expression of *Mettl7a1* significantly reduces their invasion potential (Figure 4E).

**Figure 5.**
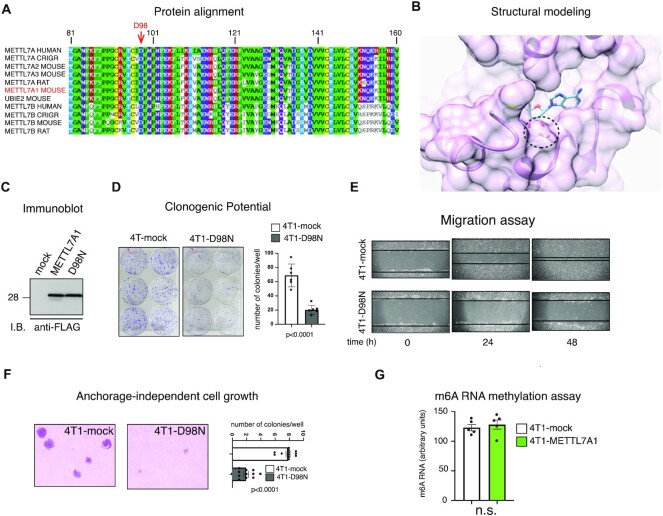
Mutation in the SAM binding domain leaves unaffected METTL7A1 function in 4T1 cells. (**A**) Multiple alignment of the putative Methyltransferase domain of human METTL7A and METTL7B with homologous rodent sequences. (**B**) Model structure of METTL7A1 together with a SAM molecule in the binding pocket. The double hydrogen bond between the SAM ribose O2 and O3 hydroxyl oxygens and the Asp98 side chain are shown as dotted red lines. (**C**) Total extracts were prepared from 4T1 cells stably transfected with either the empty vector (mock) or FLAG-tagged METTL7A1 expression vector (METTL7A1) or with the FLAG-tagged D98N mutant of METTL7A1 (D98N) and analyzed by SDS-PAGE followed by Immunoblot as indicated. The immunoblot is representative of three experiments that yielded similar results. (**D**) Either mock or 4T1-D98N cells were plated at low density in sextuplicate. After 6–10 days, colonies were stained and counted using the imaging analysis software package ImageJ 1.53a (http://imagej.nih.gov/ij, NIH). On the left a representative image of stained colonies. The values of three independent experiments performed in sextuplicate (±SEM) are plotted on the right. (**E**) Scratch wound healing assays. Scratch wounds were introduced into confluent monolayers of either 4T1-mock or 4T1-D98N cells. Cultures were photographed and the width of the wound was measured at 0, 24 and 48 h after the scratch was made. Representative images of three independent experiment performed in sextuplicate are shown. (**F**) Either 4T1-mock or 4T1-D98N were cultured in soft agar for 21 days and phase contrast micrographs were taken at 10× magnification. The number of colonies was counted and plotted. A representative image of colonies is shown on the left while the values in the bar graph (on the right) are averages (±SEM) of three independent experiments performed in sextuplicate. (**G**) Total RNA was extracted from either 4T1-mock or 4T1-METTL7A1 and m6A was quantified using the colorimetric EpiQuik m6A RNA methylation quantification kit. The values are averages (±SEM) of five independent experiments performed in duplicate. Statistical significance (Student's t test) has been calculated using GraphPad Prism 9 for macOS software and indicated.

Both human METTL7A and mouse METTL7A1 have been reported as putative methyltransferases (3,8). We wanted to investigate whether the phenotype that we observed in 4T1 upon expression of *Mettl7a1* was dependent on the predicted methyltransferase activity. Protein sequence analysis of human and rodent METTL7A1 family members (Figure 5A) revealed that they structurally belong to *Class I* methyltransferases containing the Rossmann fold which is the binding pocket for SAM (a common substrate involved in methyl group transfer) (23). Based on the modeling of the SAM binding pocket (Figure 5B), we identified Aspartic acid at position 98 (D98) as an amino acid potentially involved in METTL7A1 enzymatic activity. D98 was mutagenized to Asparagine (N) that possesses a sterically similar side chain but should significantly reduce the affinity of the protein for SAM. The mutant protein METTL7A1-D98N was stably expressed in 4T1 cells (4T1-D98N cells) (Figure 5C). 4T1-D98N cells exhibited reduced clonogenic potential, growth in soft agar, and migration with a behavior very similar to that displayed by 4T1-METTLA1 cells (Figure 5D–F). Further, considering that N6-methyladenosine (m^6^A) modification of RNA represents a very common modification operated by methyltransferases, we quantified METTL7A1-induced m6A modification of total RNA. The analysis of m^6^A-modified RNA using a colorimetric assay showed no significant difference between 4T1-METTL7A1 and mock cells (Figure 5G).

Altogether these data indicate that the expression of METTL7A1 in 4T1 cells impairs their transformation potential independently of its putative methyltransferase activity.

### METTL7A1 belongs to a multi-protein complex that includes translation factors and affects protein expression in 4T1 cells.

**Figure 6.**
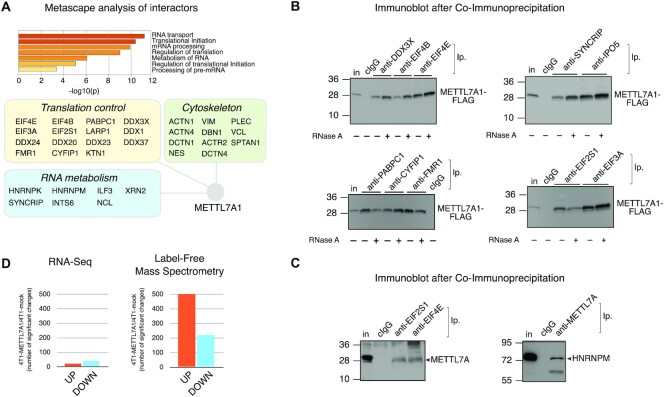
METTL7A1 belongs to a multi-protein complex that includes translation factors and affects protein expression in 4T1 cells. (**A**) Metascape analysis of METTL7A1 partners identified by MS after immunopurification of protein complexes (top panel). Interactors belonging to three major functional categories are represented in the schematic (bottom panel). (**B**) Co-immunoprecipitation of FLAG-tagged METTL7A1 with its molecular partners in 4T1 cell extracts. When indicated, cell extracts were pre-treated for 30 min at 37°C with RNase A (100 μg/ml). (**C**) Co-immunoprecipitation of endogenous METTL7A1 with its molecular partners in NMuMG cell extracts. Immunoprecipitations and immunoblots shown in panels B and C are representative of three experiments that yielded similar results. (**D**) Number of significant transcript changes based on RNA-Seq (left panel) and protein changes based on label-free MS (right panel) in 4T1-METTL7A1 cells compared to 4T1-mock cells.

To gain insight into METTL7A1 molecular functions, we performed immunoaffinity purification of proteins interacting with FLAG-tagged METTL7A1 in NMuMG-METTL7A1 cells followed by Mass Spectrometry (MS) analysis whose results were filtered through highly stringent criteria (see Materials and Methods). We selected 87 interactors (59 were absent in immunocomplexes from mock cells and 28 displayed a ≥4-fold enrichment in immunocomplexes from NMuMG-METTL7A1 when compared to mock cells). Metascape analysis (http://metascape.org) revealed enrichment in factors involved in RNA metabolism, mRNA translation, and cytoskeleton organization among METTL7A1 interactors (Figure 6A). Strikingly, the analysis of protein-protein interactions performed by using the online STRING tool (24) showed that the vast majority (∼90%) of identified METTL7A molecular partners belong to a single large cluster (Supplementary Figure S4A). Then, we performed co-immunoprecipitation experiments that confirmed METTL7A1 interaction with factors implicated in translation initiation (including EIF3A, EIF4B, EIF2S1, EIF4E, DDX3X, FMR1, CYFIP1, PABPC1) and with RNA binding proteins (including HNRNPM, IPO5 and SYNCRIP) (Figure 6B and C). The observed interactions are RNA-independent being unaffected by cell extracts treatment with RNase A (Figure 6B).

Next, we investigated whether METTL7A1 expression in 4T1 cells results in gene expression changes. First, we performed RNA-Seq analysis in 4T1-METTL7A1 compared to mock cells and this analysis revealed that METTL7A1 has a limited effect on the transcriptome of 4T1 cells (Figure 6D, left panel). In parallel, we performed MS-based label-free protein quantification in 4T1-METTL7A1 in comparison to mock cells and observed a significant number of changes in protein expression (Figure 6D, right panel). Interestingly, gene ontology analysis of the molecular function of the proteins whose levels are affected by METTL7A1 expression in 4T1 cells revealed a highly significant representation of RNA binding proteins, proteins implicated in translation initiation as well as factors involved in cell adhesion (Supplementary Figure S4B).

Altogether our data indicate that METTL7A1 interacts with several factors involved in mRNA translation and RNA metabolism and suggest that it might be involved in protein translation.

### METTL7A1 modulates translation of select genes

**Figure 7.**
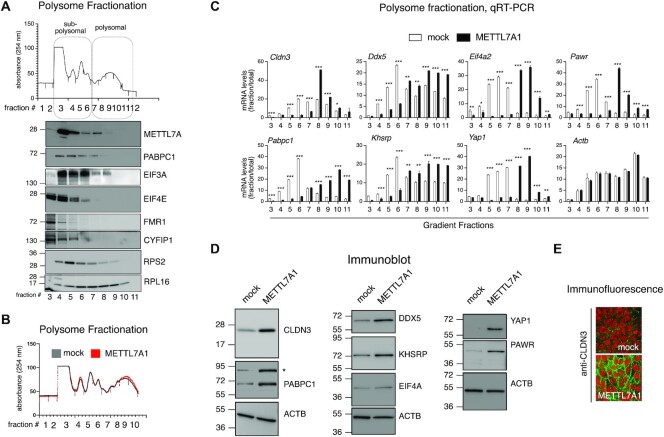
METTL7A1 modulates translation of select genes. (**A**) Representative example of polysome profiles obtained from cytoplasmic lysates of NMuMG cells fractionated by sucrose gradients. The relative absorbance of the various fractions is indicated (top panel). Immunoblot analysis of protein extracted from each gradient fraction. The identity of the proteins is indicated on the right. (**B**) Representative example of polysome profiles obtained from cytoplasmic lysates of 4T1-mock cells (mock) and 4T1-METTL7A1 cells (METTL7A1) fractionated by sucrose gradients. The relative absorbance of the various fractions is indicated. Polysome profiles are representative of at least three independent fractionations performed on biological triplicates. (**C**) qRT-PCR analysis of RNA extracted from the indicated polysome fractions and total RNA prepared from 4T1-mock cells (mock) and 4T1-METTL7A1 cells (METTL7A1). RNA levels in each fraction are presented upon normalization for total RNA levels (see Supplementary Figure S5A). The values of qRT-PCR experiments shown are averages (±SEM) of three independent experiments performed in triplicate. Statistical significance (Student's t test) has been calculated using GraphPad Prism 9 for macOS software (* *P*< 0.01, ** *P*< 0.001, *** *P*< 0.0001). (**D**) Immunoblot analysis of lysates prepared from 4T1-mock cells (mock) and 4T1-METTL7A1 cells (METTL7A1). The identity of each protein is presented on the right. Asterisk marks the position of a non-specific band. E. Immunofluorescence analysis of 4T1-METTL7A1 cells grown on chamber slides and stained with anti-CLDN3 antibody. Scale bars represent 50 μm.

We resolved polysome fractions from cytoplasmic extracts derived from NMuMG cells through sucrose gradient centrifugation (Figure 7A, top panel). Immunoblot analysis of gradient fractions revealed that METTL7A1 is mainly present in subpolysomal fractions together with some regulators of protein translation that we identified as METTL7A1 molecular partners (Figure 7A, bottom panel and see also Figure 6 and Supplementary Figure S4).

Next, we analyzed polysome profiles from cytoplasmic extracts derived from 4T1 mock and 4T1-METTL7A1 cells and observed that the expression of METTL7A1 did not alter the global profile (Figure 7B). Therefore, we hypothesized that METTL7A1 expression could affect the translation of specific mRNAs. To verify this hypothesis, we took advantage of RNA-Seq analyses performed on total RNA (see above) and on RNA purified from either sub-polysomal or polysomal fractions, as well as on our label-free MS analysis. We selected a representative number of factors whose expression is enhanced in 4T1-METTL7A1 cells at the protein level (according to the top functional categories detected in our label-free MS analysis, Supplementary Figure S4B) and analyzed by qRT-PCR the levels of the corresponding transcripts across RNA extracted from each fraction of the sucrose gradient profiles upon normalization with the respective total mRNA levels. Data shown in Figure 7C, Supplementary Figure S5A, and S5B show that METTL7A1 expression in 4T1 cells causes a shift of select mRNAs from sub-polysomal fractions to polysomal fractions. *Actb* and *Mmp14* were used as negative controls. Immunoblot and immunofluorescence analyses demonstrated that the METTL7A1-induced shift of mRNAs from subpolysomal to polysomal fractions results in increased protein levels (Figures 7D, E). Metabolic labelling of cells with L-azidohomoalanine (AHA) followed by Biotin Click-iT^®^ reaction and precipitation with Streptavidin beads supported our hypothesis that METTL7A1 favors biosynthesis of select factors (Supplementary Figure S5C shows CLND3 as an example and ACTB as a negative control). Further, analysis of proteasome activity did not reveal differences between mock and 4T1-METTL7A1 cells ruling out an effect of METTL7A1 on protein degradation (Supplementary Figure S5D).

We explored the possibility that METTL7A1 favors the association of the mRNAs shown in Figure 7C with translation factors by immunoprecipitating ribonucleoprotein complexes (RIP analysis) from either 4T1-mock or 4T1-METTL7A1 cells using antibodies directed to EIF4E, EIF2S1, EIF3 and EIF4B. As shown in Supplementary Figure S6A, the interaction between these translation factors and METTL7A1 targets is significantly enhanced in 4T1-METTL7A1 cells while the interaction with control mRNA *Actb* is unaffected.

Altogether our data show that METTL7A1 is present in subpolysomal fractions and contributes to translation of select genes likely by influencing the step of translation initiation.

## DISCUSSION

In this report we provide evidence that *Mettl7a1* gene is transcriptionally induced by the lncRNA *EPR* and modulates the translation of select mRNAs.

We were intrigued by our previous results showing that, despite a strong interaction of *EPR* with *Mettl7a1* regulatory region and a significant induction of *Mettl7a1* in NMuMG-*EPR* cells, we did not observe enhanced local levels of histone activation marks as occurs for most *EPR*-bound transcriptionally activated genes (2). Here we show that *EPR* activates *Mettl7a1* gene transcription through reconfiguration of three-dimensional (3D) chromatin architecture. Our finding allows us to hypothesize that *EPR*, by interacting with chromatin, might be instrumental for 3D chromatin reconfiguration on a genome-wide scale. Promoter-capture Hi-C experiments are currently in progress in our laboratory to verify the extent of *EPR*-dependent chromatin changes in the genome.

*Mettl7a1* shares with *EPR* the rapid downregulation of its expression upon treatment with TGF-β and its forced expression in immortalized mammary gland cells impairs the TGF-β-dependent induction of genes involved in EMT. METTL7A1 expression is downregulated in a variety of human cancers and, accordingly, it is virtually absent in murine mammary gland cancer cells including triple negative-like 4T1 cells. Interestingly, also *EPR* is barely detectable in transformed mammary gland cells (1), and our evidence that METTL7A1 expression in 4T1 cells inhibits colony forming activity, migration, ECM invasion, and growth in semisolid media suggests the possibility that METTL7A1 mediates certain cellular functions of *EPR*.

The molecular mechanism(s) underlying human METTL7A functions have never been explored in depth and information about murine METTL7A1 is missing. In this report, we provide initial characterization of METTL7A1 activity in modulating translation of select mRNAs. N^6^-methyladenosine (m^6^A) is the most prevalent internal modification existing on eukaryotic mRNA (25), and it impacts a variety of physiological events and, among them, mRNA translation efficiency (26). While this manuscript was in preparation, Yang and colleagues (27) described human METTL7A as a component of a ribonucleoprotein complex that can interfere with protein translation through m^6^A methylation of specific exosomal lncRNAs in adipocytes. Although both the exclusively cytoplasmic localization of METTL7A1 and the evidence that D98N mutant recapitulates the biological functions of the wild-type protein make unlikely a role for METTL7A1 as a m6A ‘writer’, we performed methylated RNA immunoprecipitation followed by high throughput sequencing (MeRIP-Seq) in mock and *Mettl7a1*-expressing 4T1 cells. MeRIP-Seq results revealed that METTL7A1 expression in 4T1 cells causes m6A changes in a very limited number (= 4) of transcripts (Supplementary Figure S6B). Among these, three RNAs encode proteins but the expression of none of them is affected by METTL7A1 expression in 4T1 cells. The differences between our results and Yang's paper might be ascribed to differences in the cell type as well as in the N-terminal sequence of mouse METTL7A1 and human METTL7A (see Supplementary Figure S6C). These differences could result in different cellular localization of mouse METTL7A1 compared to human METTL7A.

Based on our results showing that METTL7A1 is a cytoplasmic protein whose expression in 4T1 cells affects protein levels without substantial changes in mRNA abundance, we propose here a role for METTL7A1 as a regulator of select mRNA translation. Our hypothesis is supported by the evidence that METTL7A1 is present in ribosome fractions and associates with factors involved in the first steps of translation.

Recently the cytoplasmic component of METTL3 has been reported to promote mRNA translation through association with translation initiation factors independently of either its methyltransferase activity or interaction with downstream m^6^A ‘reader’ proteins (28). Further, Lin *et al.* demonstrate that METTL3 promotes cancer cell growth, survival, and invasion (28). Based on our results we propose that METTL7A1 restrains the tumorigenic potential of mammary gland cancer cells and this is obtained independently of m^6^A RNA modification activity.

We identified a group of mRNAs whose translation is regulated by METTL7A1, and focused only on transcripts whose translation is activated since our proteomic analysis showed that enhanced expression accounts for most protein changes induced by METTL7A1 expression. We were unable to identify any common motif in the 5’ UTR sequence of translationally regulated mRNAs, thus further studies will be required to clarify the molecular basis of mRNA translation selectivity.

## DATA AVAILABILITY

Proteomics data has been deposited to the EMBL-EBI-based PRIDE repository (https://www.ebi.ac.uk/pride/).

Project name: LncRNA EPR-induced METTL7A1 modulates target gene translation

Project accession: PXD034398

Project DOI: 10.6019/PXD034398

Experiment set GSE195823

This SuperSeries is composed of the following SubSeries:

GSE195819, Identification of a LncRNA EPR—>METTL7A1 pathway modulating translation of select genes (3C-Seq)

GSE195821, Identification of a LncRNA EPR—>METTL7A1 pathway modulating translation of select genes (Polysomes)

GSE195822, Identification of a LncRNA EPR—>METTL7A1 pathway modulating translation of select genes (Total RNA)

To review GEO accession GSE195823:

Go to https://www.ncbi.nlm.nih.gov/geo/query/acc.cgi?acc = GSE195823

Enter token ehajwsiybbmjvqp into the box

## Supplementary Material

gkac544_Supplemental_File
