# Supplementary material for: LncRNA EPR-induced METTL7A1 modulates target gene translation
Source: Nucleic Acids Res. 2022 Jun 24;50(13):7608–22. doi: 10.1093/nar/gkac544 (PMC9303270; doi:10.1093/nar/gkac544)
Supplement: gkac544_Supplemental_File [file gkac544_supplemental_file.pdf]

SUPPLEMENTARY DATA

LncRNA EPR-induced METTL7A1 modulates target gene translation

Paola Briata, Luca Caputo, Ettore Zapparoli, Elisa Marcaccini, Mario Passalacqua, Lorenzo Brondolo, Domenico Bordo, Annalisa Rossi, Chiara Nicoletti, Gabriele Bucci, Pier Lorenzo Puri, Alberto Inga, Roberto Gherzi

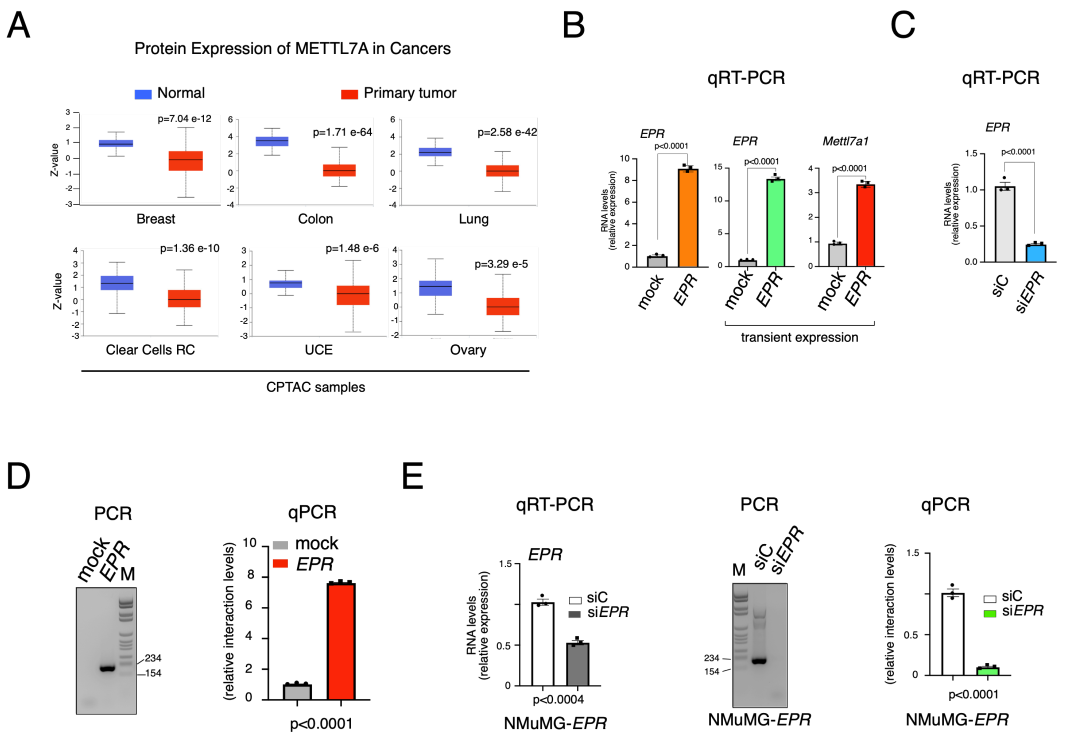

Supplementary Figure S1.

A. Expression analysis of METTL7A protein in the indicated tumors (and in the corresponding normal tissues) using the UALCAN tool which is designed to access publicly available Clinical Proteomic Tumor Analysis Consortium (CPTAC) Confirmatory/Discovery datasets. RC is for renal cell and UCE is for Uterine corpus endometrial carcinoma. B. Total RNA was extracted from either mock and NMuMG-*EPR* cells (mock, and *EPR*, respectively; left) or NMuMG cells transiently transfected with either empty vector (mock) or a plasmid expressing *EPR* (*EPR*) (middle and right) and analyzed by qRT-PCR as indicated; C. total RNA was extracted from NMuMG cells transiently transfected with either control siRNA (siC) or si*EPR* and analyzed by qRT-PCR as indicated. D. PCR (left) and qPCR (right) analysis of DNA fragments obtained upon the first *DpnII* digestion and ligation during 3C experiments. The DNA was derived from either NMuMG-mock cells (mock) or NMuMG-EPR cells (EPR) as indicated. E. NMuMG-EPR were transiently transfected with either control siRNA (siC) or siRNA designed to silence EPR expression (siEPR). The expression of EPR was analyzed by qRT-PCR (left panel). 3C analysis was performed on either siC- or siEPR- silenced NMuMG-EPR cells and PCR (middle) or qPCR (right) analysis of DNA fragments obtained upon the first *DpnII* digestion and ligation was performed. The values of qRT-PCR experiments shown are averages (±SEM) of three independent experiments performed in triplicate. Statistical significance (Student's t test) has been calculated using GraphPad Prism 9 for macOS and indicated. 3C-PCR analyses were conducted on biological duplicates and representative gels are shown.

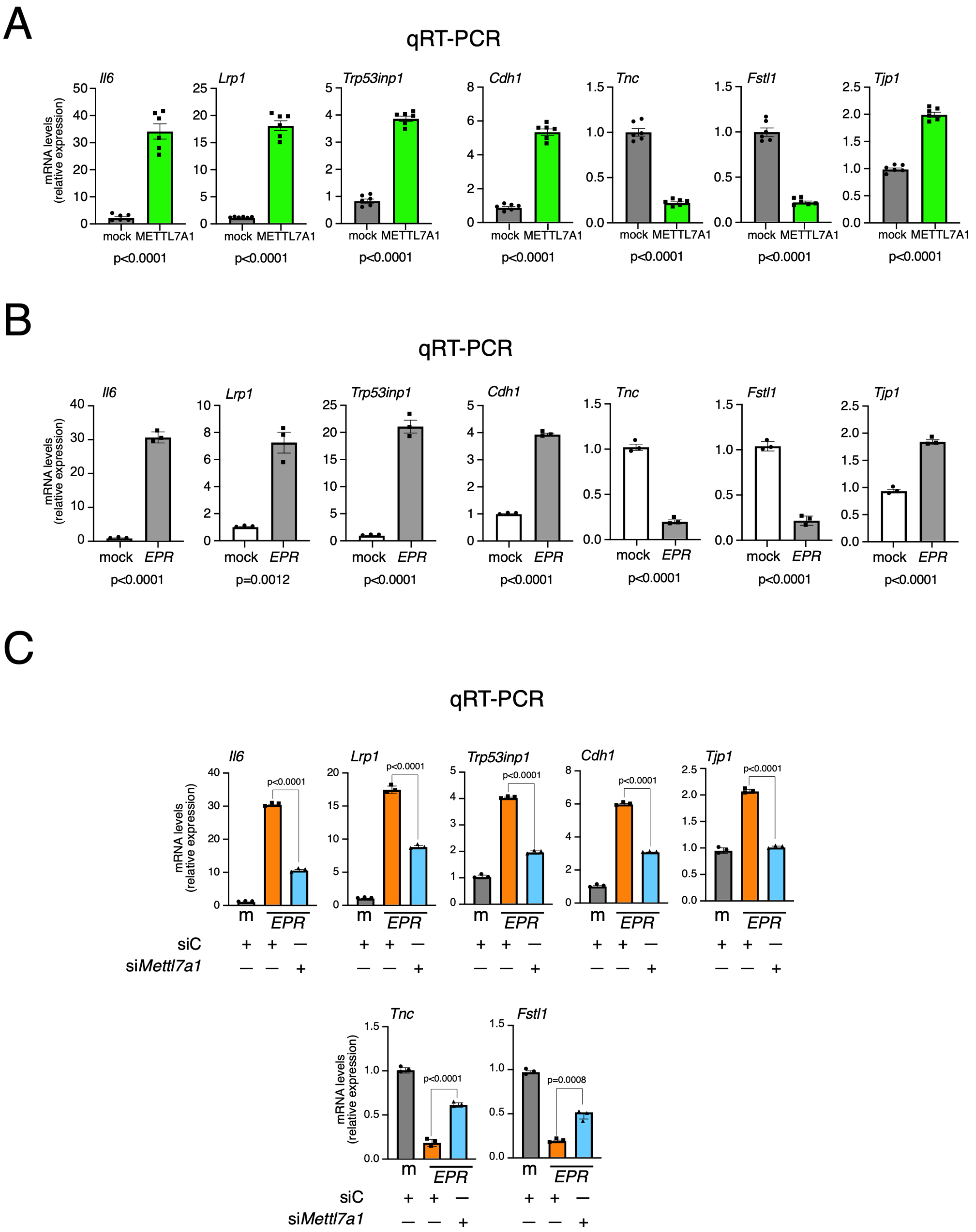

Supplementary Figure S2.

A. Total RNA was extracted from mock and NMuMG-METTL7A1 (METTL7A1) cells and analyzed by qRT-PCR using the indicated primers. B. Total RNA was extracted from mock and NMuMG-*EPR* (*EPR*) cells and analyzed by qRT-PCR using the indicated primers. C. Total RNA was extracted from mock (m) and NMuMG-*EPR* (*EPR*) cells transiently transfected with either control siRNA (siC) or si*Mettl7a1* and analyzed by qRT-PCR as indicated. The values of qRT-PCR experiments shown are averages (±SEM) of three independent experiments performed in triplicate. Statistical significance (Student's t test) has been calculated using GraphPad Prism 9 for macOS and indicated.

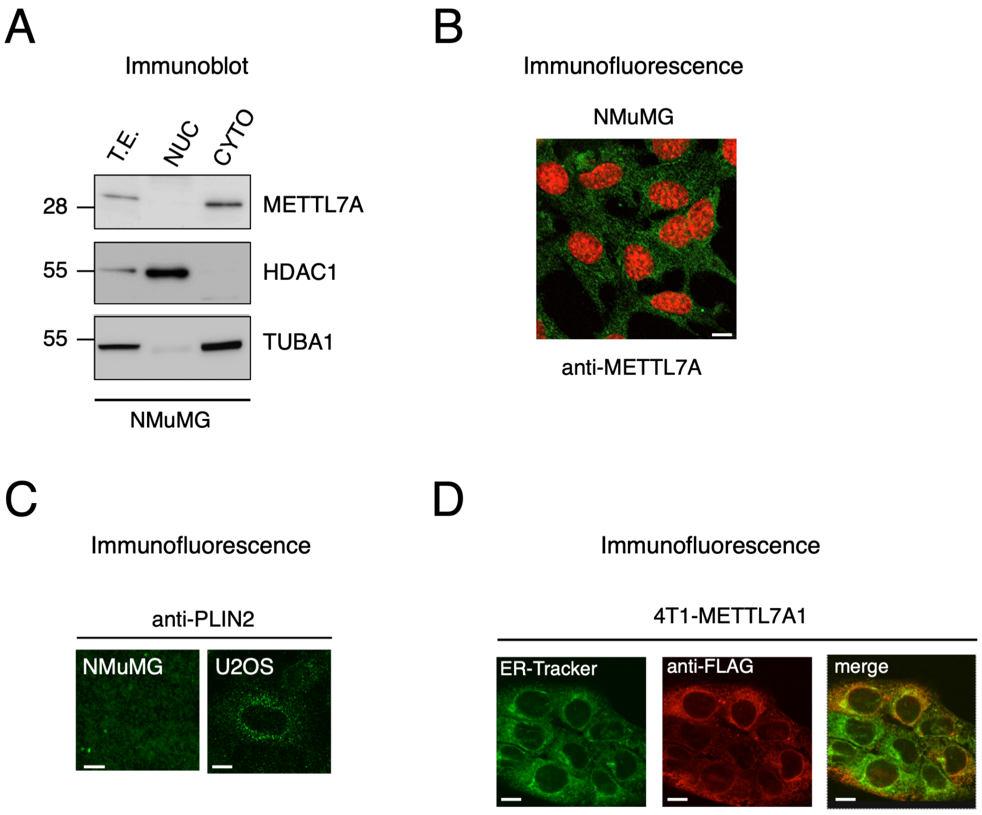

<u>Supplementary Figure S3</u>.

A. Total, cytoplasmic, and nuclear extracts were prepared from NMuMG cells and analyzed by SDS-PAGE followed by Immunoblot using the indicated antibodies. B. Immunofluorescence analysis of NMuMG cells grown on chamber slides and stained with anti-METTL7 antibody. Anti-METTL7A staining is in green while nuclear counterstaining with TO-PRO-3 is in red. C. Immunofluorescence analysis of NMuMG cells (left) and U2OS cells (right) with anti-PLIN2 antibody. D. Immunofluorescence analysis of 4T1-METTL7A1 cells with ER-Tracker (green, left) and anti-FLAG antibody (red, middle). The merge of the two stainings is presented (yellow) on the right. Scale bars represent 50 µm.

A

STRING predicted protein-protein interaction

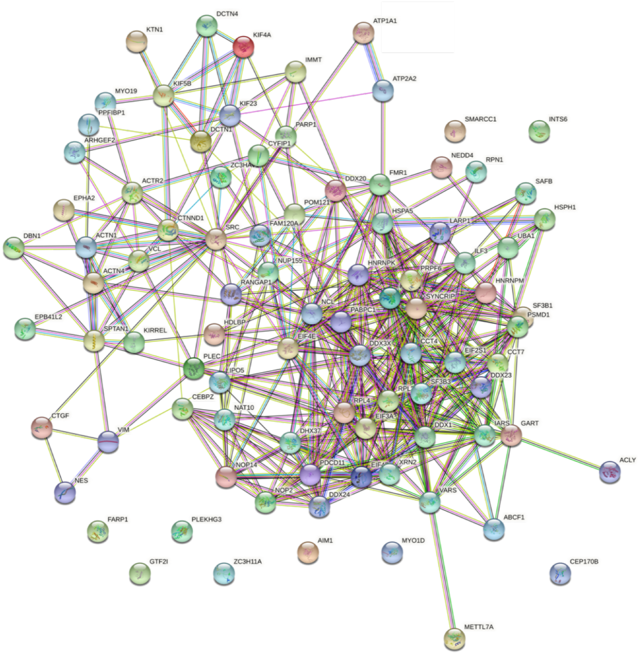

B

Gene Ontology Molecular Function

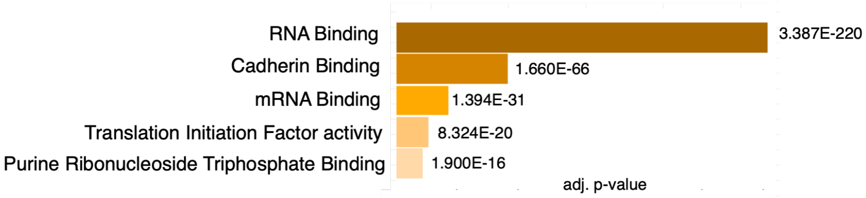

Supplementary Figure S4.

A. Analysis of known and predicted protein-protein interactions of METTL7A with its molecular partners identified by MS analysis in NMuMG-METTL7A1 using the STRING database. B. Gene ontology molecular function analysis (using the online EnrichR tool) of proteins whose levels are affected by METLL7A1 expression in 4T1 cells as identified by label-free MS analysis.

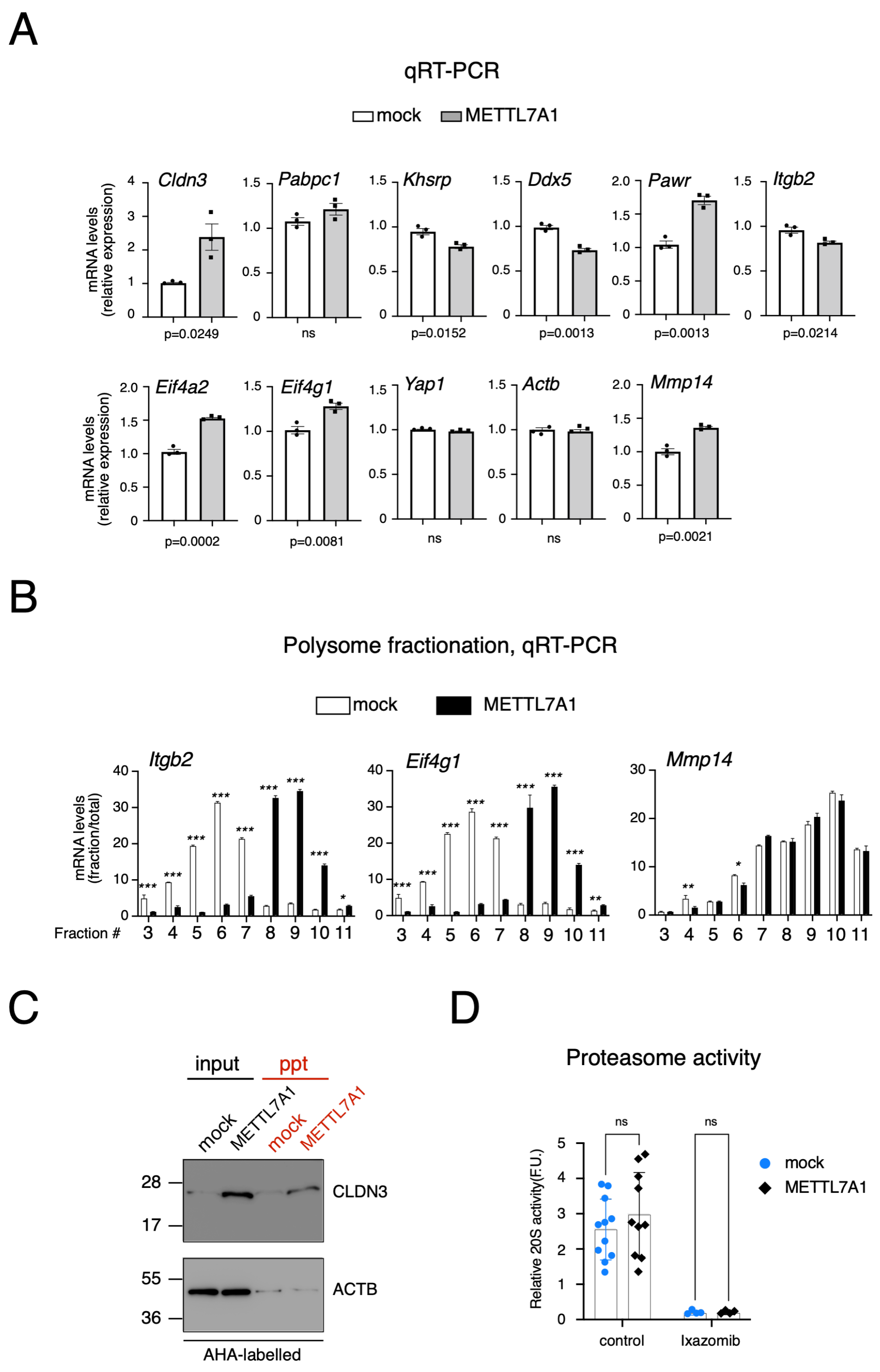

Supplementary Figure S5.

A. Total RNA was extracted from 4T1 cells stably transfected with either the empty vector (mock) or METTL7A1 (METTL7A1) and analyzed by qRT-PCR as indicated. B. qRT-PCR analysis of RNA extracted from the indicated polysome fractions and total RNA prepared from 4T1-mock cells (mock) and 4T1-METTL7A1 cells (METTL7A1). RNA levels in each fraction are presented upon normalization for total RNA levels (see panel A of this Figure). The values

of qRT-PCR experiments shown are averages (±SEM) of three independent experiments performed in triplicate. Statistical significance (Student’s t test) has been calculated using GraphPad Prism 9 for macOS. C. Either 4T1-mock or 4T1-METTL7A1 cells were labelled with AHA for 210 minutes, extracts were prepared and “Clicked” to Biotin and purified by Streptavidin beads precipitation as described under Materials and Methods. Both input and precipitated material (ppt) were analyzed by SDS-PAGE and Immunoblot with the indicated antibodies. The immunoblots displayed are representative of three experiments that yielded similar results. D. Relative proteasome activity expressed as relative Fluorescence Units in 4T1-mock and 4T1-METTL7A1 cells either treated with Ixazomib or left untreated (control). Data are averages (±SD) of three independent experiments. Statistical significance (2way Anova with Sidak’s multiple comparisons test) has been calculated using GraphPad Prism 9 for macOS and indicated, ns = not significant.

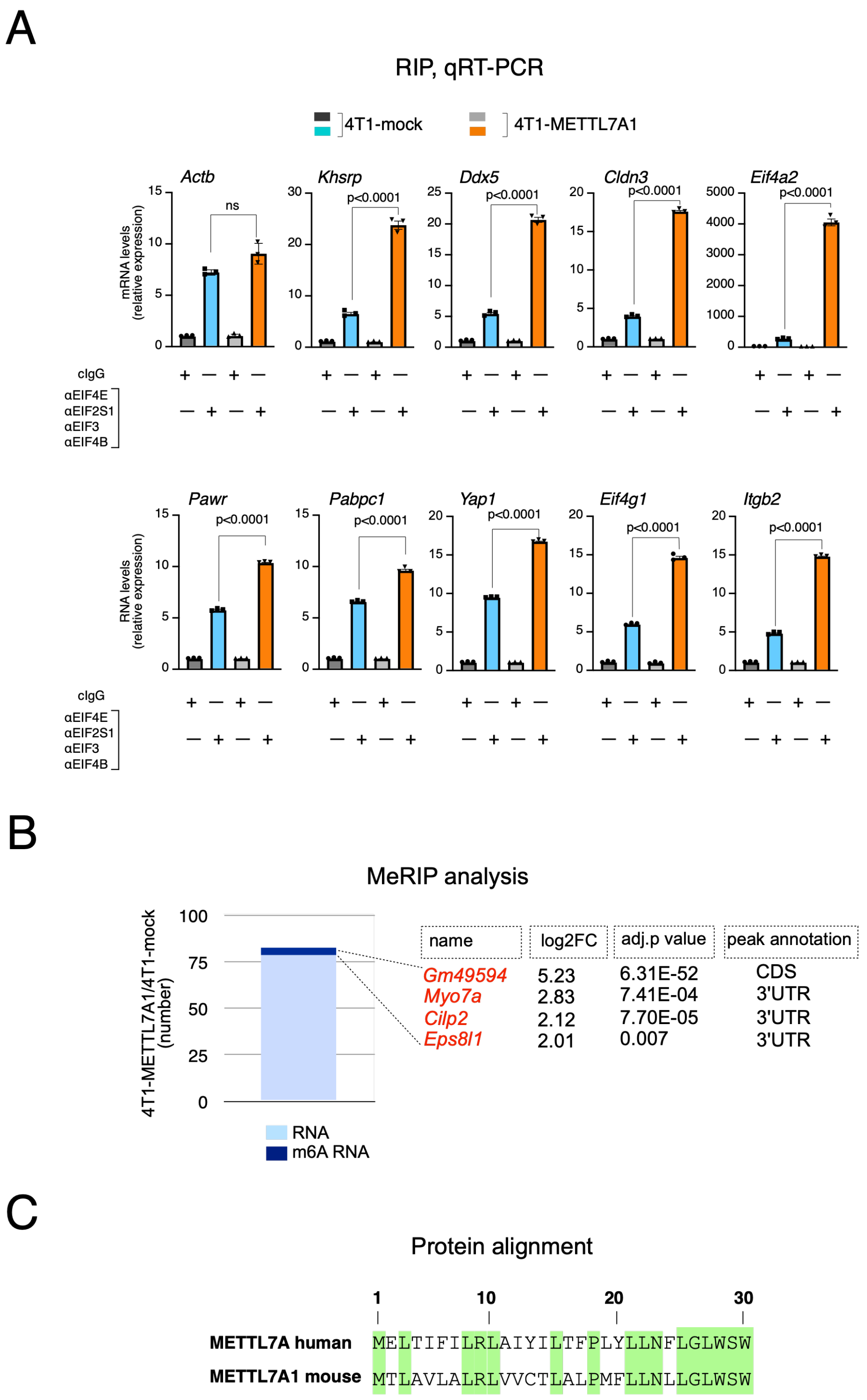

Supplementary Figure S6.

A. Total extracts were prepared from either 4T1-mock or 4T1-METTL7A1 cells and immunoprecipitated using either rabbit control IgG (cIgG) or a mixture of antibodies directed to EIF4E, EIF2S1, EIF3, and EIF4B. RNA was prepared from immunocomplexes, retrotranscribed and analyzed by qPCR using the indicated primers. The values shown are averages (±SEM) of three independent experiments performed in triplicate. Statistical significance (Student's t test) has been calculated using GraphPad Prism 9 for macOS and

indicated. B. Total RNA was extracted from 4T1 cells stably transfected with either the empty vector (mock) or METTL7A1 (METTL7A1) and subjected to MeRIP analysis. The number of significantly (log2FCl ≥1.5 and p value < 0.01) upregulated transcrips (light blue) or upregulated m^6A-containing RNAs (and their identity) in 4T1-METTL7A1 cells compared to 4T1-mock cells is indicated. C. Alignment of the N-terminal domain of human METTL7A and murine METTL7A1.

Supplementary Table

S1.

List of primary antibodies used for in this study.

ANTIBODY	SUPPLIER	CATALOGUE #
METTL7A	Abcam	ab79207
METTL7A (mouse monoclonal)	Origene	TA809878
ACTB	Sigma	AC-74
HDAC1	Santa Cruz	sc-7872
TUBA1	Sigma	DM1
FLAG TAG	Sigma	F1804
CDH1	Santa Cruz	sc-31020
TJP1	Abcam	ab96587
CALNEXIN	Sigma	C4731
SMAD3	Abcam	ab28379
EIF2S1	Sigma	SAB4300523
EIF3A	Sigma	HPA038315
CYFIP1	Sigma	SAB2700152
FMR1	Sigma	F4055
SYNCRIP	Sigma	041265
IPO5	Sigma	SAB4200179
DDX3X	Sigma	HPA001648
EIF4B	Sigma	AV40359
EIF4E	Sigma	SAB2100666
HNRNPM	Origene	TA803306
PABPC1/C3	Millipore	ABE40
MYC TAG	Millipore	06-549
RPS2	Sigma	SAB2701183
CLDN3	Sigma	SAB4500435
DDX5	Abcam	ab10261
EIF4A2	Sigma	HPA068286
YAP1	Merck	ZRB1001
PAWR	Sigma	HPA012640
TNC	Origene	TA354535
ATF3	Millipore	MABN124
PLIN2	Sigma	HPA016607

Supplementary Table S2.

List of primers used for qRT-PCR, for constructing 3C-Seq libraries, and for 3C-PCR/qPCR analyses.
The Illumina adapter sequences of primers used for 3C-Seq analysis are highlighted.

Primers for qRT-PCR	Forward	Reverse
mmu.Rpl32	CTGGCCATCAGAGTCACCAA	TGCACACAAGCCATCTACTCA
mmu.EPR	AACTGCGCAACCTAGTCCTG	GCTCCATGGAAGACTAACGG
mmu.Pabpc1	AATATGCCCGGTGCTATCCG	GACTCGTGGAACCTGTGAGG
mmu.Pawr	TTACTAATGCGTGCGGCCTT	AGCATGTGGAAAGAGGAGACG
mmu.Itgb2	CCCTCTGCAAACACAACGTG	GTCGGAAGACAAGTCCCTGG
mmu.Fn1	GCAGGAAAGTCACCCAGACA	CTGTGGGAGGGGTGTTTGAA
mmu.Eif4a2	GCCTCAGCAAGCAATCATCC	GGCCAGACCACCTATATAAAGTGAA
mmu.Eif4g1	ACCCTGCTGGATCCCAAGTA	ACATATGTGGAACGCCCCTG
mmu.Yap1	GGGGGTTCTCTGGCTAACAC	AACCAACGTAAGAGCAGCGA
mmu.Mmp14	TTCGTGTTGCCTGATGACGA	GCTTATCTGGGACAGAGGGC
mmu.Cldn3	TCGAAGGGCAGTTGATTCCC	ACATGGCTGCTGGACTTGAA
mmu.Khsrp	CCTGATTTTGGCTTTGGGGG	CCAGTTTCTTGCTGTCTGGC
mmu.Ddx5	GCCCAAGAGCTGTGTGCACTGT	TCCTGACTTCCAGTTGTGGCT
mmu.Actb	GCAAGCAGGAGTACGATGAGT	AGGGTGTAAAACGCAGCTCAG
mmu.Fstl1	ACGCTCCCACCTTCGCCTCT	GTCACCAGCGAGAGCGCCAG
mmu.Zeb1	AGCCTTAAGGAAGCAGCCAG	ACATCAACACTGGTCGTCCC
mmu.Mettl7a1	CTGACGAGAGAGAGCTGGAAG	GCCTGGATGTGCTGTAGCTTTA
mmu.Zeb2	GTCTCTGCAAGTGCCATCCT	ACTGACACGGGTGCTTCAAA
mmu.Cdh2	CGAGAGGCCTATCCATGCTG	CCCAATATCCCCAGGGTGTG
mmu.Snai2	CATCCTTGGGGCGTGTAAGT	ATGGCATGGGGGTCTGAAAG
mmu.Ocln	TGGCAAGCGATCATACCCAG	AGGAATCTCCTGGGCCACTT
mmu.Tjp1	GGACACCAAAGCATGTGAGC	AGGGTAAGGCATTCCTGCTG
mmu.Tnc	AACGGACTGCCCACATCTCA	TTCCGGTTCAGCTTCTGTGGTA
mmu.Lrp1	TCACCAACCCAGTGTATGCC	AGCAGTTCTCGCTTCTCGTC
mmu.Trp53inp1	TAGCTTTGCCCCTACTCCCT	GCCAAACAAATTCTGACGGCT
mmu.Cdh1	AACCCAAGCACGTATCAGGG	ACTGCTGGTCAGGATCGTTG
mmu.Il6	CCCCAATTTCCAATGCTCTCC	CGCACTAGGTTTGCCGAGTA

Primers for 3C-Seq	P5 (see ref. 13)	P7 (see ref.13)
P5Mettl7a1	AATGATACGGCGACCACCGAGATCTACACTCTTTCCCTACACGACGCTCTTCCGATCTCAGCAACCACATGGTGGCTCA	
P7-701Mettl7a1 (mock)		CAAGCAGAAGACGGCATACGAGATTCGCCTTAGTCTCGTGGGCTCGGAGATGTGTATAAGAGACAGATGTTCCTGGTTGGTTGGG
P7-702Mettl7a1		CAAGCAGAAGACGGCATACGAGATCTAGTACGGTCTCGTGGGCTCGGAGATGTGTATAAGAGACAGATGTTCCTGGTTGGTTGGG
Primers for 3C-PCR analysis	Forward	Reverse
mmu.VP3(Viewpoint)	CCTGCCTCTGTCAGTTCTGG	
mmu.Atf1 locus (3C)		TACTAAGGTCTCGGGCGGT
mmu.Actb locus (3C)		CTAACCTAGTAAAGCTGTCTGGTGT
